# MCM3AP-AS1: An Indispensable Cancer-Related LncRNA

**DOI:** 10.3389/fcell.2021.752718

**Published:** 2021-10-07

**Authors:** Xiao Yu, Qingyuan Zheng, Qiyao Zhang, Shuijun Zhang, Yuting He, Wenzhi Guo

**Affiliations:** ^1^Department of Hepatobiliary and Pancreatic Surgery, The First Affiliated Hospital of Zhengzhou University, Zhengzhou, China; ^2^Key Laboratory of Hepatobiliary and Pancreatic Surgery and Digestive Organ Transplantation of Henan Province, The First Affiliated Hospital of Zhengzhou University, Zhengzhou, China; ^3^Open and Key Laboratory of Hepatobiliary & Pancreatic Surgery and Digestive Organ Transplantation at Henan Universities, Zhengzhou, China; ^4^Henan Key Laboratory of Digestive Organ Transplantation, Zhengzhou, China

**Keywords:** long non-coding RNA, MCM3AP-AS1, biological function, regulatory mechanism, biomarker

## Abstract

Long non-coding RNAs (lncRNAs) are a class of RNA molecules with transcripts longer than 200 nucleotides that have no protein-coding ability. MCM3AP-AS1, a novel lncRNA, is aberrantly expressed in human cancers. It is significantly associated with many clinical characteristics, such as tumor size, tumor-node-metastasis (TNM) stage, and pathological grade. Additionally, it considerably promotes or suppresses tumor progression by controlling the biological functions of cells. MCM3AP-AS1 is a promising biomarker for cancer diagnosis, prognosis evaluation, and treatment. In this review, we briefly summarized the published studies on the expression, biological function, and regulatory mechanisms of MCM3AP-AS1. We also discussed the clinical applications of MCM3AP-AS1 as a biomarker.

## Introduction

Cancer is a fatal disease that is often caused by somatic mutations (Kennedy et al., 2019; Andrei et al., 2020; Costa et al., 2020). Genomic alterations can lead to a series of malignant features, including cell migration, invasion, and metastasis. Conventional cancer therapies, including surgery, radiotherapy, and chemotherapy, have limitations (Liang et al., 2021). Molecular targeted therapy, a new therapeutic approach, overcomes these limitations and has advantages in cancer treatment (Ethier et al., 2021).

Long non-coding RNAs (lncRNAs) are a class of RNA molecules with transcript lengths longer than 200 nucleotides that have no protein-coding ability (Thum, 2014; Lorenzen and Thum, 2016; Liu et al., 2017; Xu et al., 2020; Jin et al., 2021). The function of lncRNAs is related to their special subcellular localization. The lncRNAs located in the nucleus participate in gene regulation at the epigenetic and transcription levels. Moreover, lncRNAs in the cytoplasm are involved in interactions with proteins in the cytoplasm and the regulation of the metabolism of mRNAs, such as endogenous competitive RNAs (ceRNAs), which interact with microRNAs. Increasing evidence indicates that lncRNAs are important modulators of different biological functions (Zehendner et al., 2020). The overexpression of lncRNA OTUD6B-AS1 inhibits cell proliferation, migration, and invasion in clear cell renal cell carcinoma (Wang et al., 2019a). Elevated levels of lncRNA H19 decreased sensitivity to tamoxifen in breast cancer (Wang et al., 2019b). Moreover, lncRNAs have been reported to function via multiple signaling pathways in cancer progression. LNRRIL6 promotes cancer progression by activating the IL-6/STAT3 pathway in colorectal cancer (CRC) (Wang et al., 2019c). Long non-coding RNA EPB41L4A-AS2 inhibits cell proliferation and migration by downregulating miR-301a-5p expression and upregulating FOXL1 expression in hepatocellular carcinoma (HCC) (Wang et al., 2019f).

**FIGURE 1:**
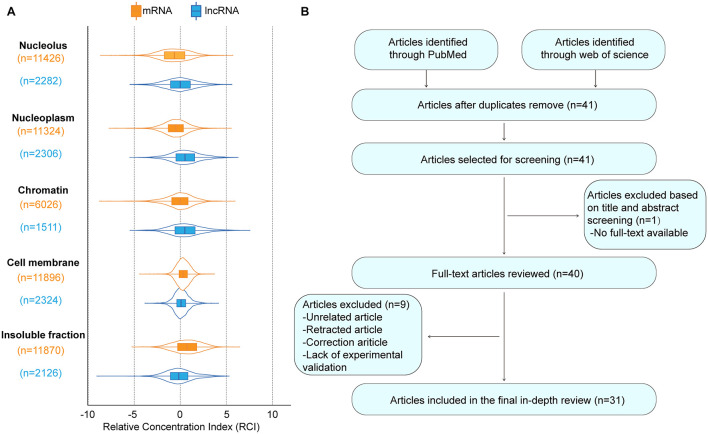
The basic information of MCM3AP-AS1. **(A)** The subcellular localization plot of MCM3AP-AS1 displayed by lncATLAS database. For K562 cells, subnuclear and sub-cytoplasmic fractions were available and Relative Concentration Index (RCI) for all lncRNAs and mRNAs was computed. Boxplot shows the distribution of these RCI values for each subnuclear and sub-cytoplasmic compartment (“n” indicates total number of genes in a distribution). **(B)** Flow diagram of the research search and selection process.

MCM3AP-AS1 is located in 46,228,977-46,259,390 of chromosome 21, and the subcellular localization of MCM3AP-AS1 is chromatin and nucleoplasm (Figure 1A). MCM3AP-AS1 was found to be dysregulated in a variety of cancers, including breast cancer, CRC, gastric cancer, HCC, and prostate cancer (PCa). MCM3AP-AS1 has great potential for use in cancer diagnosis, prognosis evaluation, and treatment. In this review, we first summarized the expression profile of MCM3AP-AS1 and the cellular processes in which MCM3AP-AS1 is involved. Then, we clarified the mechanism of MCM3AP-AS1 in two parts*: in vitro* cell experiments and *in vivo* experiments. The former part enabled superficial function verification, while the latter enabled further confirmation of the potential regulatory mechanism of MCM3AP-AS1. Briefly, we outline the role of lncRNA MCM3AP-AS1 in tumorigenesis by integrating recent research findings.

## Evidence Acquisition

We carried out exhaustive research employing PubMed and the Web of Science database to seek articles up to August 2021 using the keywords MCM3AP-AS1, MCM3AP-AS, MCM3APAS, tumor, cancer, and carcinoma. We assessed all results according to the titles and abstracts and selected articles related to our theme. All unrelated articles, letters, meeting proceedings, correction articles, and retracted articles were excluded. After this, the full text of any selected article was reviewed independently by two authors. Figure 1B shows a flow diagram of the study selection process.

## Expression and Biological Functions of MCM3AP-AS1 in Human Cancer

The expression levels of MCM3AP-AS1 are significantly dysregulated in human cancers (Table 1). MCM3AP-AS1 plays a vital role in the occurrence and development of various cancers. Its expression is significantly associated with several clinical characteristics. Moreover, *in vitro* assays have shown that it markedly promotes or suppresses tumor progression by controlling cell biological functions. In this section, we discussed the emerging roles of MCM3AP-AS1 in different cancers (Table 2).

**TABLE 1:** Expression and associated clinical features of the lncRNA MCM3AP-AS1 in cancer.

Type	Expression	Feature	References
Breast cancer	upregulated	tumor estrogen receptor expression, and tumor progesterone receptor expression	Riahi et al., 2021
Burkitt Lymphoma	upregulated	tumor size, tumor stage, and poor prognosis	Guo et al., 2020
Cervical squamous cell carcinoma	downregulated	poor survival	Lan et al., 2020
Clear cell renal cell carcinoma	upregulated	/	Wang et al., 2019a
Colorectal cancer	upregulated	poor survival	Ma et al., 2020
Colorectal cancer	downregulated	poor prognosis, higher TNM stage, tumor size, and CEA level	Dai et al., 2021
Colorectal cancer	upregulated	poor survival	Zhou et al., 2021
Endometrioid carcinoma	upregulated	/	Yu J. et al., 2021
Hepatocellular carcinoma	upregulated	large tumor size, high tumor grade, advanced tumor stage, and poor prognosis	Wang et al., 2019e
Hepatocellular carcinoma	/	overall survival	Zhang et al., 2019
Nasopharyngeal carcinoma	upregulated	poor survival	Sun et al., 2020
Lung cancer	upregulated	/	Li et al., 2020b
Small cell lung cancer	upregulated	survival rate	Luo et al., 2021
Non-small cell lung cancer	upregulated	/	Shen et al., 2021
Oral squamous cell carcinoma	upregulated	poor prognosis	Hou et al., 2020
Oral squamous cell carcinoma	upregulated	/	Li and Jiang, 2020
Pancreatic cancer	upregulated	survival rates	Yang et al., 2019
Prostate cancer	upregulated	overall survival	Jia et al., 2020
Prostate cancer	upregulated	pathological stage, Gleason score, and AR expression	Li et al., 2020a
Prostate cancer	/	overall survival	Chen et al., 2021
Prostate cancer	upregulated	disease–free survival	Wu et al., 2020

**TABLE 2:** The biological functions and molecular mechanisms of MCM3AP-AS1.

Type	Expression	Function	Related genes	References
Breast cancer	upregulated	cell proliferation, migration, and invasion	miR-28-5p, and CENPF	Chen et al., 2020
Breast cancer	upregulated	cell proliferation	miR-708-5p	Riahi et al., 2021
Burkitt lymphoma	upregulated	cell viability, apoptosis, and chemoresistance	miR-15a, and EIF4E	Guo et al., 2020
Cervical squamous cell carcinoma	downregulated	cell proliferation	miR-93	Lan et al., 2020
Colorectal cancer	upregulated	cell cycle	miR-545, and CDK4	Ma et al., 2020
Clear cell renal cell carcinoma	upregulated	proliferation, inflammation, Pro-angiogenesis	E2F1, DPP4	Wang et al., 2019a
Colorectal cancer	downregulated	cell proliferation, and migration	miR-19a-3p, and FOXF2	Dai et al., 2021
Colorectal cancer	upregulated	cell proliferation, colony formation, migratory, and invasive ability	miR-193a-5p, and SENP1	Zhou et al., 2021
Colorectal cancer	/	/	miR-599, and ARPP19	Yu Y. et al., 2021
Endometrioid carcinoma	upregulated	invasion, and migration	miR-708-5p	Yu J. et al., 2021
Gastric cancer	upregulated (CDDP resistance)	CDDP resistance	miR-138, and FOXC1	Sun et al., 2021
Gastric cancer	upregulated	cell proliferation, and apoptosis	miR-708-5p	H Wang et al., 2020
Glioblastoma	upregulated	cell viability, migration, tube formation of GECs, and angiogenesis	miR-211, KLF5, and AGGF1	Yang et al., 2017
Hepatocellular carcinoma	upregulated	cell proliferation, colony formation, cell cycle progression, and induced apoptosis	miR-194-5p, FOXA1, and FOXA1 restoration	Wang et al., 2019e
Hepatocellular carcinoma	/	invasion, and HDLECs	miR-455, and EGFR	Zhang et al., 2019
Lung cancer	upregulated	cell proliferation, migration, and angiogenesis	YY1, miR-340-5p, and KPNA4	Li et al., 2020b
Small cell lung cancer	upregulated	invasion, and migration	miR-148a, and ROCK1	Luo et al., 2021
Non-small cell lung cancer	upregulated	proliferation, migration and invasion	miR-195-5p, and E2F	Shen et al., 2021
Nasopharyngeal carcinoma	upregulated	cell proliferation, and apoptosis	miR-34a	Sun et al., 2020
Oral squamous cell carcinoma	upregulated	proliferation, migration and invasion	miR-363-5p	Hou et al., 2020
Oral squamous cell carcinoma	upregulated	proliferation, migration and invasion	miR-204-5p, and FOXC1	Li and Jiang, 2020
Pancreatic cancer	upregulated	proliferation, migration, and invasion	miR-138-5p, and FOXK1	Yang et al., 2019
Papillary thyroid cancer	upregulated	proliferation, migration, and invasion	miR-211-5p, and SPARC	Liang et al., 2019
Prostate cancer	upregulated	proliferation, and invasion	miR-543-3p, SLC39A10, and PTEN	Jia et al., 2020
Prostate cancer	upregulated	proliferation, invasion, migration, and apoptosis	DNMT1, DNMT3, NPY1R, and MAPK	Li et al., 2020a
Prostate cancer	/	bone metastasis	/	Chen et al., 2021
Prostate cancer	upregulated	proliferative ability, and apoptosis	miR-876-5p, WNT5A, and WNT5A	Wu et al., 2020

### Breast Cancer

Breast cancer is one of the most common types of malignancy in women worldwide (Liang et al., 2020; Carreira et al., 2021; Han et al., 2021). The levels of lncRNA MCM3AP-AS1 are significantly upregulated in breast cancer tissues and cell lines (Chen et al., 2020; Riahi et al., 2021). The level of MCM3AP-AS1 was positively associated with estrogen receptor (ER) and progesterone receptor (PR) expression, whereas no significant differences were observed between MCM3AP-AS1 and HER2 expression profiles in breast cancer patients. Functionally, MCM3AP-AS1 affected cell biological functions to control breast cancer progression by regulating specific pathways. MCM3AP-AS1 knockout inhibited the proliferation, invasion, and migration of breast cancer cell lines. These findings may facilitate the development of novel therapeutics for breast cancer.

### Colorectal Cancer

**FIGURE 2:**
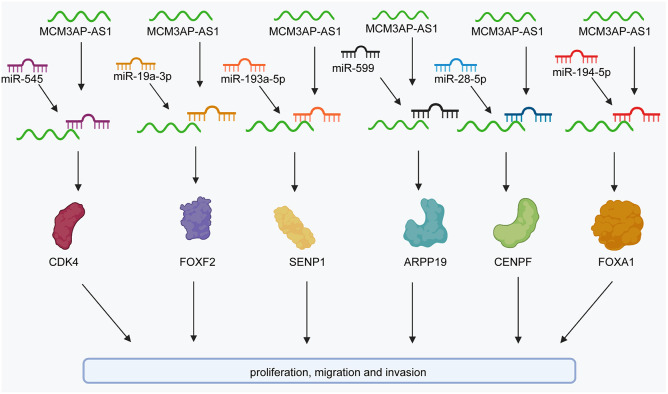
The regulatory molecular mechanisms of the lncRNA MCM3AP-AS1 in CRC, breast cancer, and liver cancer. MCM3AP-AS1 facilitates the expression of SENP1 to attenuate cell proliferation and colony formation by sponging miR-193a-5p in CRC. MCM3AP-AS1 promotes the expression of FOXF2 to inhibit cell proliferation by sponging miR-19a-3p in CRC. It also promotes the cell cycle by activating the miR-545/CDK4 pathway. MCM3AP-AS1 inhibits ARPP19 levels by sponging miR-599 in colorectal cancer.

Colorectal cancer (CRC) is one of the most prevalent cancers and a leading cause of cancer-related death worldwide (Oki et al., 2016; Wang et al., 2019d; Yarla et al., 2019; Zhao et al., 2021). Some studies have revealed that MCM3AP-AS1 expression is markedly upregulated in CRC tissues compared to corresponding normal tissues (Ma et al., 2020; Zhou et al., 2021). In terms of prognosis, MCM3AP-AS1 levels are negatively associated with overall survival (OS). Functionally, elevated MCM3AP-AS1 expression promotes cell proliferation, colony formation, migration, and invasion and arrests the cell cycle at the G1 phase in CRC cell lines (Ma et al., 2020; Zhou et al., 2021) (Figure 2). In contrast, Dai et al. found that MCM3AP-AS1 expression was decreased in CRC tissues (Dai et al., 2021). The expression of MCM3AP-AS1 was positively correlated with OS in CRC patients. Moreover, the levels of MCM3AP-AS1 were negatively associated with tumor-node-metastasis (TNM) stage, tumor size, and carcinoembryonic antigen (CEA) levels in CRC. Functionally, MCM3AP-AS1 significantly reduced the proliferation and migration of CRC cells (Figure 2). The results were validated by siRNA knockdown experiments. However, MCM3AP-AS1 expression needs to be further studied in CRC. Further evidence-based basic and clinical studies are needed to increase the evidence base.

### Gastric Cancer

Gastric cancer is the fifth most common malignancy and the second leading cause of cancer-related mortality worldwide (Liu et al., 2016; Seidlitz et al., 2019; Kang et al., 2020; Harada et al., 2021; Li et al., 2021). Cisplatin (CDDP) is a well-known chemotherapeutic agent used to treat gastric cancer (Germann et al., 2002; Ivanova et al., 2013; Huang et al., 2019). Cisplatin resistance is the main reason for the poor therapeutic effects in gastric cancer (Wang et al., 2018; Zhang Q. et al., 2020). The expression levels of MCM3AP-AS1 were evidently upregulated in MGc-803, SGC-7901, NCI-N87 (NCI-N87/CDDP), and AGS cells (AGS/CDDP) (Wang et al., 2020; Sun et al., 2021). *In vitro* evidence suggested that the levels of MCM3AP-AS1 are positively associated with the half-maximal inhibitory concentration (IC_50_) of CDDP in gastric cancer. MCM3AP-AS1 was also found to facilitate cell proliferation, migration, and invasion and decrease CDDP sensitivity in gastric cancer cell lines (Sun et al., 2021).

### Liver Cancer

Hepatocellular carcinoma (HCC) is a common primary malignancy of the liver and typically occurs in patients with underlying chronic liver disease (Degasperi et al., 2020; Goh et al., 2020; Yip et al., 2020; Zhou et al., 2020; Xia et al., 2021). MCM3AP-AS1 expression is upregulated in HCC tissues and cell lines (Wang et al., 2019e). The levels of MCM3AP-AS1 are positively associated with tumor size, stage, and grade in HCC patients. Increased MCM3AP-AS1 expression was associated with a worse prognosis in HCC (Wang et al., 2019e; Zhang et al., 2019). *In vitro* cell experiments suggested that silencing MCM3AP-AS1 inhibited the formation of human lymphatic endothelial cells in HCC. Moreover, MCM3AP-AS1 significantly promotes the proliferation, colony formation, cell cycle progression, and metastasis of HCC cells (Wang et al., 2019e; Zhang et al., 2019).

### Oral Squamous Cell Carcinoma

Oral squamous cell carcinoma (OSCC), a head and neck cancer, seriously affects the quality of life of affected patients, both psychologically and physically (Ng et al., 2017; Bray et al., 2018; Almangush et al., 2020). Hou et al. revealed that MCM3AP-AS1 expression was elevated in both OSCC cells and tissues and that a high expression level of MCM3AP-AS1 was correlated with a poor prognosis in OSCC patients. Moreover, overexpression of MCM3AP-AS1 could enhance the proliferation, migration, and invasion of OSCC cells. Inhibiting MCM3AP-AS1 had the opposite effect on the above cell events (Hou et al., 2020). Li et al. found similar phenomena in OSCC cells and found different molecular mechanisms than Hou, which would be elaborated in a later section (Li and Jiang, 2020). Therefore, we have adequate evidence to indicate that MCM3AP-AS1 has the potential to act as a biomarker for OSCC patients.

### Prostate Cancer

Prostate cancer (PCa) is the most commonly diagnosed cancer and the second leading cause of cancer-related death in Americans (Amarasekera et al., 2019; Berger et al., 2019; Ge et al., 2020). The expression levels of MCM3AP-AS1 were markedly increased in PCa tissues and cell lines (Jia et al., 2020; Li et al., 2020a; Wu et al., 2020). MCM3AP-AS1 levels were negatively correlated with OS in PCa patients (Jia et al., 2020; Chen et al., 2021).

A negative association has also been reported between MCM3AP-AS1 and disease-free survival in PCa (Wu et al., 2020). MCM3AP-AS1 expression correlates with the Gleason score, pathological stage, and androgen receptor expression in PCa (Li et al., 2020a). MCM3AP-AS1 knockdown inhibited cell proliferation, migration, and invasion and promoted the apoptosis of PCa cell lines (Jia et al., 2020; Li et al., 2020a; Wu et al., 2020). In addition, MCM3AP-AS1 expression was significantly associated with bone metastases in PCa (Chen et al., 2021).

### Lung Cancer

Lung cancer (LC) continues to be one of the most frequent cancers worldwide. The number of patients and deaths related to LC has continued to increase in recent years (Schwartz and Cote, 2016; Jones and Baldwin, 2018; Bade and Dela Cruz, 2020). MCM3AP-AS1 was elevated in small cell lung cancer (SCLC), and a high expression level of MCM3AP-AS1 was accompanied by a low survival rate. MCM3AP-AS1 overexpression could facilitate the migration and invasion of SCLC cells (Luo et al., 2021). In non-small-cell lung cancer (NSCLC), MCM3AP-AS1 was also obviously upregulated. MCM3AP-AS1 also could promote the proliferation, invasion, and migration of NSCLC cells. Li et al. generally found that MCM3AP-AS1 could accelerate angiogenesis, in addition to cell proliferation and migration, in LC. In conclusion, MCM3AP-AS1 had the potential to act as a biomarker in LC (Li et al., 2020b).

### Reproductive System Cancers

For females, the incidence and motility of reproductive system cancers are the highest among cancers worldwide, indicating that they seriously threaten women’s health (Torre et al., 2017; Hernandez-Silva et al., 2020). Interestingly, MCM3AP-AS1 participates in the progression of most reproductive system cancers, including cervical cancer and endometrial cancer. For endometrioid carcinoma (EC), MCM3AP-AS1 expression was upregulated in cancer tissues compared with adjacent normal tissues. Overexpression of MCM3AP-AS1 increased the migration and invasion rate of EC cells. Both migration and invasion were inhibited when MCM3AP-AS1 was knocked down (Yu J. et al., 2021). However, Lan et al. demonstrated that MCM3AP-AS1 expression was reduced in cervical squamous cell carcinoma (CSCC) and that MCM3AP-AS1 overexpression significantly inhibit the proliferation of CSCC cells (Lan et al., 2020). These results suggest that MCM3AP-AS1 plays dual roles in reproductive tumors, and the underlying mechanism is probably worth studying.

### Other Cancers

MCM3AP-AS1 was also found to be upregulated in Burkitt lymphoma, glioblastoma, LC, nasopharyngeal carcinoma, clear cell renal cell carcinoma, pancreatic cancer, and papillary thyroid cancer tissues compared to the corresponding normal tissues (Yang et al., 2017, 2019; Liang et al., 2019; Guo et al., 2020; Li et al., 2020b; Qiu et al., 2020; Sun et al., 2020). The MCM3AP-AS1 expression profile was positively associated with tumor size and stage in Burkitt lymphoma. Higher levels of MCM3AP-AS1 indicate a worse prognosis in Burkitt lymphoma, nasopharyngeal carcinoma, and pancreatic cancer. Further *in vitro* experiments confirmed that MCM3AP-AS1 expression was upregulated in pancreatic cancer cell lines (PANC-1, BxPC-3, MIA PaCa-2, Capan-2, and AsPC-1). MCM3AP-AS1 inhibited cell proliferation and migration in LC, pancreatic cancer, and papillary thyroid cancer. It also promoted tumor angiogenesis in glioblastoma and LC and increased cell viability in Burkitt lymphoma and glioblastoma.

In contrast to the above findings, MCM3AP-AS1 expression is markedly downregulated in CSCC tissue samples and predicts a poor outcome. Overexpression of MCM3AP-AS1 reduces CSCC cell proliferation, and MCM3AP-AS1 acts as a tumor suppressor during CSCC development and progression.

In conclusion, MCM3AP-AS1 expression was markedly elevated in breast cancer (Chen et al., 2020; Riahi et al., 2021), Burkitt lymphoma (Guo et al., 2020), gastric cancer (Wang et al., 2020; Sun et al., 2021), glioblastoma (Yang et al., 2017), HCC (Wang et al., 2019e), LC (Li et al., 2020b), nasopharyngeal carcinoma (Sun et al., 2020), pancreatic cancer (Yang et al., 2019), papillary thyroid cancer (Liang et al., 2019), and PCa (Li et al., 2020a; Wu et al., 2020). However, MCM3AP-AS1 expression was downregulated in CSCC patients (Lan et al., 2020) (Table 1). Collectively, MCM3AP-AS1 has the potential to act as a prognostic biomarker for many cancers.

## Regulatory Molecular Mechanisms of MCM3AP-AS1 in Human Cancer

From the aforementioned studies, we can conclude that MCM3AP-AS1 plays an important role in the regulation of various biological functions, such as cell growth, motility, cell cycle, drug resistance, and angiogenesis. In this section, we summarized the regulatory molecular mechanisms of MCM3AP-AS1 in cancer, both *in vivo* and *in vitro.*

### *In vitro* Cell Experiment

#### Cell Growth

**FIGURE 3:**
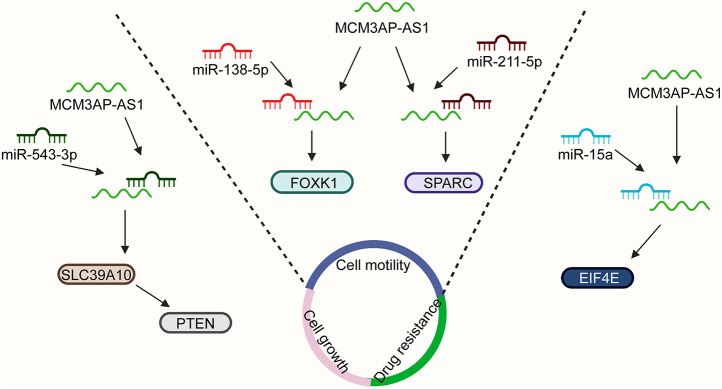
MCM3AP-AS1 functions by interacting with specific molecular pathways. MCM3AP-AS1 enhances cell proliferation via the miR-543-3p/SLC39A10/PTEN axis in PCa. MCM3AP-AS1 reduces the levels of miR-138-5p and increases the expression of FOXK1 to promote cell migration in pancreatic cancer. Cell invasion is enhanced by the activation of the miR-211-5p/SPARC pathway in papillary thyroid cancer. MCM3AP-AS1 regulates the sensitivity of lymphoma cells to doxorubicin by regulating the miR-15a/EIF4E pathway.

MCM3AP-AS1 facilitates cell proliferation by reducing miR-708-5p levels in breast cancer cells (Riahi et al., 2021). MCM3AP-AS1 facilitates the expression of SENP1 to attenuate cell proliferation and colony formation by sponging miR-193a-5p in CRC cells (Zhou et al., 2021). It also inhibited gastric cancer cell proliferation and promoted apoptosis by downregulating miR-708-5p levels (Wang et al., 2020). In HCC, MCM3AP-AS1 regulates cellular processes, such as cell proliferation, cell cycle progression, and cell apoptosis by activating the miR-194-5p/FOXA1 pathway (Wang et al., 2019e). MCM3AP-AS1 enhances the proliferation of PCa cells by the miR-543-3p/SLC39A10/PTEN (Figure 3) and miR-876-5p/WNT5A pathways in PCa (Jia et al., 2020; Li et al., 2020a). Additionally, it activates the MAPK pathway to induce cell proliferation by promoting methylation of the NPY1R promoter in PCa (Wu et al., 2020). Silencing of MCM3AP-AS1 suppresses KPNA4 expression to impair cell proliferation by acting as a sponge of miR-340-5p in LC (Li et al., 2020b). MCM3AP-AS1 does not affect the levels of miR-34a, whereas elevated miR-34a expression suppresses cell proliferation by downregulating MCM3AP-AS1 expression in nasopharyngeal carcinoma (Sun et al., 2020). Silencing MCM3AP-AS1 expression inhibits cell proliferation and colony formation by regulating the miR-138-5p/FOXK1 axis in pancreatic cancer (Yang et al., 2019) and plays the same role by controlling the miR-211-5p/SPARC pathway in papillary thyroid cancer (Liang et al., 2019). In contrast, MCM3AP-AS1 markedly downregulates the expression of miR-93 and inhibited cell proliferation in CSCC (Lan et al., 2020). Some researchers have found that MCM3AP-AS1 promotes the expression of FOXF2 to enhance cell proliferation by sponging miR-19a-3p in CRC (Dai et al., 2021).

#### Cell Motility

Cell motility is a physiological process that is required for embryonic development, wound healing, immune surveillance, and cancer metastasis (Tojkander et al., 2015; Swaminathan et al., 2016; Cummins et al., 2018). MCM3AP-AS1 functions as a sponge of miR-193a-5p to upregulate SENP1 expression and facilitate cell migration and invasion in CRC (Zhou et al., 2021). Overexpression of MCM3AP-AS1 enhances cell migration and invasion by regulating DNMT1/DNMT3 (A/B) methylation-mediated overexpression of NPY1R and the miR-543-3p/SLC39A10/PTEN pathway in PCa (Jia et al., 2020; Li et al., 2020a). MCM3AP-AS1 expression is mediated by YY1 and promotes the upregulation of KPNA4, which facilitated the migration of LC cells by sponging miR-340-5p (Li et al., 2020b). LncRNA MCM3AP-AS1 reduces the levels of miR-138-5p and increased the expression of FOXK1, promoting cell migration in pancreatic cancer (Yang et al., 2019) (Figure 3). Cell proliferation and invasion are enhanced by the activation of the miR-211-5p/SPARC pathway in papillary thyroid cancer (Liang et al., 2019).

#### Angiogenesis and Drug Resistance

Angiogenesis, the formation of new blood vessels from existing vessels, plays a critical role in physiological and pathological conditions (Ramjiawan et al., 2017; Li et al., 2019). Aberrant angiogenesis can support the metabolism of tumors and contribute to tumor progression (Cebulla et al., 2014; Wu et al., 2014; Mao et al., 2015). The lncRNA MCM3AP-AS1 accelerated tumor angiogenesis by targeting the miR-211/KLF5/AGGF1 pathway in glioblastoma (Yang et al., 2017). The elevated expression of MCM3AP-AS1 facilitates angiogenesis by regulating the miR-340-5p/KPNA4 axis in LC (Li et al., 2020b). Resistance to chemotherapy is the main cause of chemotherapy failure in cancers (Si et al., 2019; Gao et al., 2020; Jena and Mandal, 2021). *In vitro* evidence suggests that increased MCM3AP-AS1 controls the sensitivity of lymphoma cells to doxorubicin by regulating the miR-15a/EIF4E pathway (Guo et al., 2020) (Figure 3). MCM3AP-AS1 reduces gastric cancer cell sensitivity to cisplatin by regulating the miR-138/FOXC1 pathway (Sun et al., 2021). The results may provide novel ideas for targeted therapy of lymphoma and gastric cancer.

### Experiments *in vivo*

The results of the *in vitro* experiments were further confirmed by experiments using animal models *in vivo*. In a nude mouse model of CRC, MCM3AP-AS1 expression was positively associated with tumor growth, tumor weight, and the number of lung metastatic tumor nodules (Zhou et al., 2021). MCM3AP-AS1 promotes tumor growth by activating the miR-28-5p/CENPF pathway in breast cancer *in vivo* (Chen et al., 2020). Furthermore, MCM3AP-AS1 enhanced the levels of FOXA1 to suppress tumorigenesis by sponging miR-194-5p in an HCC xenograft model (Wang et al., 2019e). Moreover, MCM3AP-AS1 silencing decreased the volume of PCa and inhibited the expression of SLC39A10 in BALB/c mice (Jia et al., 2020). MCM3AP-AS1 also contributed to PCa progression via regulation of the MAPK/NPY1R axis *in vivo* (Li et al., 2020a). Animal experiments have demonstrated that downregulation of MCM3AP-AS1 contributes to the expression of miR-15a and PARP, whereas it inhibits the expression of Mcl-1 and EIF4E in lymphoma (Guo et al., 2020). MCM3AP-AS1 significantly promotes tumor growth by activating the miR-211-5p/SPARC pathway in papillary thyroid cancer (Liang et al., 2019).

*In vivo* and *in vitro*, MCM3AP-AS1 has various biological functions, including cell proliferation, colony formation, migration, invasion, and chemoresistance. Interestingly, the mechanisms related to MCM3AP-AS1 are all similar and involve the ceRNA network. For researchers in related fields, this idea is worthy of reference.

## MCM3AP-AS1 as a Biomarker and Treatment Target in Cancer

Cancer prognosis monitoring is critical for reducing cancer-related deaths. Dysregulated expression patterns of MCM3AP-AS1 have great value for the diagnosis and prognosis of cancer. The levels of lncRNA MCM3AP-AS1 are negatively associated with OS of CRC patients (Zhou et al., 2021). MCM3AP-AS1 levels are also negatively correlated with OS in PCa, papillary thyroid cancer, and nasopharyngeal carcinoma (Liang et al., 2019; Jia et al., 2020; Sun et al., 2020; Chen et al., 2021). In addition, receiver operating characteristic (ROC) analyses showed that the specificity and sensitivity values of MCM3AP-AS1 were 0.58 and 0.76, respectively, in breast cancer patients (Riahi et al., 2021). Therefore, MCM3AP-AS1 can be regarded as a potential diagnosis and prognosis biomarker in multiple cancers.

Treatment of cancer using molecular-targeted therapy is a promising strategy. MCM3AP-AS1 is a novel molecular target for cancer therapy. MCM3AP-AS1 regulates cancer progression through a series of pathways, such as miR-194-5p/FOXA1 (Wang et al., 2019e), miR-138-5p/FOXK1 (Yang et al., 2019), miR-211-5p/SPARC (Liang et al., 2019), and miR-15a/EIF4E (Guo et al., 2020). And the miR-194-5p/FOXA1 axis is further confirmed *in vivo* experiments through constructing HCC xenograft model. MCM3AP-AS1 knockdown inhibits cell proliferation and colony formation in CRC (Yu Y. et al., 2021). Knockdown of MCM3AP-AS1 suppresses cell proliferation, migration, and invasion and promotes apoptosis in PCa cells and decreases the volume of PCa in BALB/c mice (Jia et al., 2020; Li et al., 2020a; Wu et al., 2020). MCM3AP-AS1 knockdown increases gastric cancer cell sensitivity to cisplatin (Sun et al., 2021). Silencing miR-708-5p attenuates the inhibition of cell proliferation caused by MCM3AP-AS1 in gastric cancer (Wang et al., 2020). The upregulation of NPY1R inhibits the function of MCM3AP-AS1 by inactivating the MAPK pathway in PCa (Li et al., 2020a).

## Conclusion and Future Perspectives

MCM3AP-AS1 is aberrantly expressed in human cancers, such as breast cancer, CRC, gastric cancer, HCC, and PCa. Its expression is significantly associated with several clinical characteristics. The levels of MCM3AP-AS1 are significantly associated with tumor size, TNM stage, pathological grade, and prognosis in different cancers. Additionally, it markedly promotes or suppresses tumor progression by controlling the biological functions of cells. For example, MCM3AP-AS1 upregulation promotes cell proliferation, colony formation, migration, and invasion and arrests the cell cycle at the G1 phase in CRC. MCM3AP-AS1 also plays an important role by interacting with specific molecules through a ceRNA mechanism. MCM3AP-AS1 facilitates proliferation by regulating the miR-193a-5p/SENP1, miR-543-3p/SLC39A10/PTEN, and miR-876-5p/WNT5A pathways.

**FIGURE 4:**
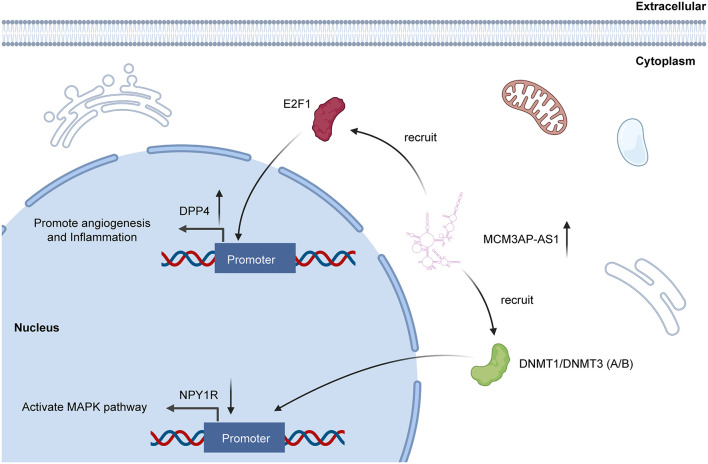
MCM3AP-AS1 functions through directly interacting with proteins. MCM3AP-AS1 enhances methylation of NPY1R promoter through recruitment of DNMT1/DNMT3 (A/B), thereby downregulating NPY1R to activate the MAPK pathway. MCM3AP-AS1 is also shown to promote the E2F1 enrichment at the DPP4 promoter, increasing DPP4 expression.

Beyond participating in ceRNA network, lncRNA can also interact with protein directly (Ferre et al., 2016). And RNA binding protein immunoprecipitation (RIP) and RNA pull-down technology could verify the interactions between lncRNAs and protein (Bierhoff, 2018). As for MCM3AP-AS1, recent researches have revealed that MCM3AP-AS1 can directly interact with special proteins and further effect the biological functions of several cancers. Qiu et al. demonstrated that MCM3AP-AS1 could interact with E2F1 and enhance the enrichment of E2F1 at the DPP4 promoter, increasing the expression of DPP4. As a result, MCM3AP-AS1 promoted angiogenesis and inflammation in clear cell renal cell carcinoma (Qiu et al., 2020). Another study also revealed that MCM3AP-AS1 can recruit DNMT1/DNMT3 (A/B) to induce methylation of NPY1R promoter. In this way, MCM3AP-AS1 decreased NYP1R expression (Li et al., 2020a) (Figure 4). The interaction between lncRNA and protein is recently proposed molecular mechanism of lncRNA. However, for MCM3AP-AS1, researches related with this mechanism are still limited. Therefore, to explore the interaction between MCM3AP-AS1 and protein is an indispensable idea for future researchers.

Exosome is a kind of extracellular vesicles secreted by cells. Exosome exerts biological functions through transporting DNA, RNA, protein, and liquid among cells (Zhang Y. et al., 2020). And these components of exosomes could play a role in receptor cells to accomplish intercellular communication (Sun et al., 2018). Recent studies revealed that lncRNA could act as component of exosome and participate in the initiation and progression of cancers. Lang et al. demonstrated that gliomas could secrete exosomes containing lncRNA POU3F3 to promote the angiogenesis (Lang et al., 2017). Similar phenomenon was observed in breast cancer. LncRNA metastasis-associated lung adenocarcinoma transcript 1 (MALAT1) was upregulated in BC, and MALAT1 was transported by exosomes to accelerate BC cell proliferation (Zhang et al., 2018). More importantly, as long as released from cells, exosomes could enter circulatory system and be isolated from available body fluid for detection (Dong et al., 2019). Thus, it is probably a crucial research direction to further explore the roles of exosome lncRNA in human tumors.

Moreover, MCM3AP-AS1 is a promising biomarker for cancer diagnosis, prognosis evaluation, and treatment. However, there is a need for additional basic and clinical experimental results before these findings can be applied in the clinic. The process of drug development is difficult and challenging. At present, RNA drugs for some diseases have been successfully listed (Crooke et al., 2018). The types of RNA drugs considered feasible include oligonucleotides, mRNA, and RNA related small molecules. Considering the drug targeting and toxicity, oligonucleotide is a promising strategy and avenue for implementing gene therapy. The advantages of oligonucleotide lie in the convenience and efficient design. Delivering oligonucleotides directly in saline solution may maintain toxicity at a low level. Chemical modifications are also feasible methods to control toxicity and reduce off-target effects. Thus, oligonucleotides are a potential strategy for drug research and development (Roberts et al., 2020). Further drug research and development can refer to our point of view.

## Author Contributions

WG, YH, and SZ designed the review. XY, QZe, and QZa wrote this manuscript. XY searched the articles and made figures. All authors worked collaboratively on the work presented here, read, and approved the final manuscript.

## Conflict of Interest

The authors declare that the research was conducted in the absence of any commercial or financial relationships that could be construed as a potential conflict of interest.

## Publisher’s Note

All claims expressed in this article are solely those of the authors and do not necessarily represent those of their affiliated organizations, or those of the publisher, the editors and the reviewers. Any product that may be evaluated in this article, or claim that may be made by its manufacturer, is not guaranteed or endorsed by the publisher.
